# Family-oriented support in genetic counselling: a scoping review of clinical practice and psychotherapeutic interventions

**DOI:** 10.1038/s41431-026-02165-z

**Published:** 2026-07-13

**Authors:** Claire E. Green, Joanna Smith, Hilary Piercy

**Affiliations:** 1 National Ehlers Danlos Syndrome Service, Sheffield Children’s Hospital Foundation Trust, Sheffield, UK & Sheffield Hallam University, Sheffield, UK; 2 The Centre for Applied Health and Social Care Research, Sheffield Hallam University, Sheffield, UK; 3 Sheffield Children’s Hospital Foundation Trust, Sheffield, UK

**Keywords:** Genetic counselling, Health care

## Abstract

Families living with inherited genetic conditions (IGCs) face practical and emotional challenges that affect individual well-being and family relationships. Although genetic counselling recognises the familial nature of genomic risk, support is often limited to patient-led disclosure. This scoping review examined how family-oriented support is conceptualised, delivered, and evaluated within genetic counselling and related psychosocial services. We define family-oriented support as any practice that explicitly considers relatives and family relationships, and systemic family-oriented care as family-systems-based models treating the family as the unit of care. Following PRISMA-ScR guidance, seven databases were searched to August 2025 for publications describing practices or interventions with a clear family element in services for people living with IGCs. Data were charted descriptively and synthesised using Rolland’s Family Systems Illness/Family Systems Genetic Illness (FSI/FSGI) frameworks. Thirty-three publications were included, describing (1) clinical practice with a family element (*n* = 12), mainly strategies to promote intrafamilial communication, and (2) psychotherapeutic approaches (*n* = 21), ten of which had been formally evaluated. Qualitative evidence suggested perceived psychosocial benefits, whereas quantitative findings were modest or mixed, constrained by small samples and limited follow-up. Most activity reflected relatively early, proband-centred positions on the FSI/FSGI continuum; only a few multifamily and narrative interventions and one local service model approximated systemic family-oriented care and remained small-scale and specialist. Work focused largely on hereditary breast/ovarian cancer, with marked gaps for untreatable or unpredictable conditions, men, partners, children, and non-Western groups. Advancing systemic, family-level care will require embedding FSI/FSGI principles in training and service design and investing in longitudinal, family-level evaluation.

## Introduction

Genomic information is both deeply personal and intrinsically familial, with one person’s test results often carrying implications for multiple relatives. Families affected by inherited genetic conditions (IGCs) frequently experience interconnected emotional and practical challenges that shape both personal well-being and family functioning. These include navigating uncertainty about when and how a condition will manifest, coping with grief related to actual or anticipated losses, and integrating genetic risk into the family’s shared identity [1–4].

As genomic testing becomes increasingly embedded in mainstream healthcare, these challenges will affect a growing number of families, prompting calls for genetic counselling approaches that engage with the family as a unit rather than the individual alone [5–8].

The canonical definition of genetic counselling emphasises helping people ‘understand and adapt to the medical, psychological, and familial implications of genetic risk information’ [9]. Commentators argue that genetic counsellors (GCs), by virtue of their counselling and communication skills training, are uniquely positioned to address familial implications of test results and to support the family as a whole [10, 11]. In practice, however, genetic counselling remains largely proband-centred, constrained by biomedical priorities, limited time, and increasing service demand [7, 8, 12–14]. The extent to which *family-oriented* support is conceptualised or operationalised within clinical genetics therefore remains unclear.

A substantial body of research has examined family communication about IGCs as a central mechanism of adaptation. Meta-syntheses demonstrate that parents often experience difficulties sharing genetic information with children—balancing emotions, gauging readiness, and ensuring understanding [15, 16]. Conversely, open communication is associated with greater family cohesion and improved psychosocial adjustment for both parents and children [1–3, 16]. Despite this, interventions to enhance family communication have shown modest or mixed effects: reviews report small gains in behaviours such as cascade testing, but limited improvement in the *quality* of communication per se [17].

Traditional ‘sender–receiver’ models of family communication, which position the patient as an information conduit, have been criticised for oversimplifying family dynamics [17, 18]. Communication is rarely linear or time-limited; it is shaped by family structure, roles, boundaries, and shared beliefs. This individual focus may inadvertently increase family conflict, delay risk communication, and miss opportunities for collective coping and meaning-making [1–4, 17]. Consequently, more systemic, family-systems-informed approaches have been proposed to better capture the relational processes involved. Rolland’s Family Systems Illness (FSI) framework [19] and its extension, the Family Systems Genetic Illness (FSGI) framework [20], conceptualise illness and genetic risk as stressors affecting the family system over time. These frameworks direct attention to the interaction between: (1) illness/genetic-condition features (e.g. onset, severity, predictability, actionability, course); (2) family-system variables (e.g. communication, roles, beliefs, cohesion, family life-cycle stage); and (3) psychosocial context (e.g. resources, culture, healthcare environment). They also emphasise that adaptation is dynamic, with different challenges arising across phases such as awareness of risk, testing, post-test adjustment, chronicity and, where relevant, loss or terminal phases.

Against this backdrop, we conducted a scoping review to map how family-oriented support is described and delivered in genetic counselling and other settings that provide support for families living with IGCs. We use *family-oriented support* as an umbrella term for any practice that explicitly considers relatives in the provision of genetic counselling or related care. We use *systemic family-oriented care* more specifically for models that conceptualise and engage the family as the unit of care and work actively with interactional patterns (e.g. roles, communication, shared meanings) across time and illness phases.

*Specifically, we asked*:

1. *How do genetic healthcare professionals (GHCPs) provide family support in clinical genetic practice and related settings?*
2. *What psychotherapeutic approaches with a family element have been described, and to what extent have they been implemented within routine clinical practice?*
3. *What is known about the acceptability and effectiveness of these approaches, particularly in supporting family communication and family adaptation?*

In planning the review, we organised these questions around three domains—conceptualisation, implementation, and evaluation of family-oriented support. During analysis, we drew on Rolland’s FSI/FSGI frameworks to interpret how far included practices and interventions moved from proband-centred risk communication toward systemic family-oriented care.

## Materials and methods

This scoping review followed JBI guidance and the PRISMA-ScR checklist to ensure a transparent and systematic approach [21] (see Supplementary Material 2). At the time of planning (2021/2022), there was no widely adopted, mandatory registry for scoping reviews in genetic counselling/clinical genetics. A protocol was developed and followed, but it was not prospectively registered.

### Search strategy

With support from an academic librarian, we developed a Population–Concept–Context (PCC)–based search strategy informed by key texts. Comprehensive searches were conducted in PubMed/MEDLINE, PsycINFO, Embase, CINAHL, Web of Science, Scopus and Google Scholar (for grey literature and citation chaining) in March 2022 and updated in August 2025. Searches were limited to English-language, human studies. The full electronic search strategy for all databases is provided in Supplementary Material 1.

### Eligibility criteria and study selection

**Fig. 1:**
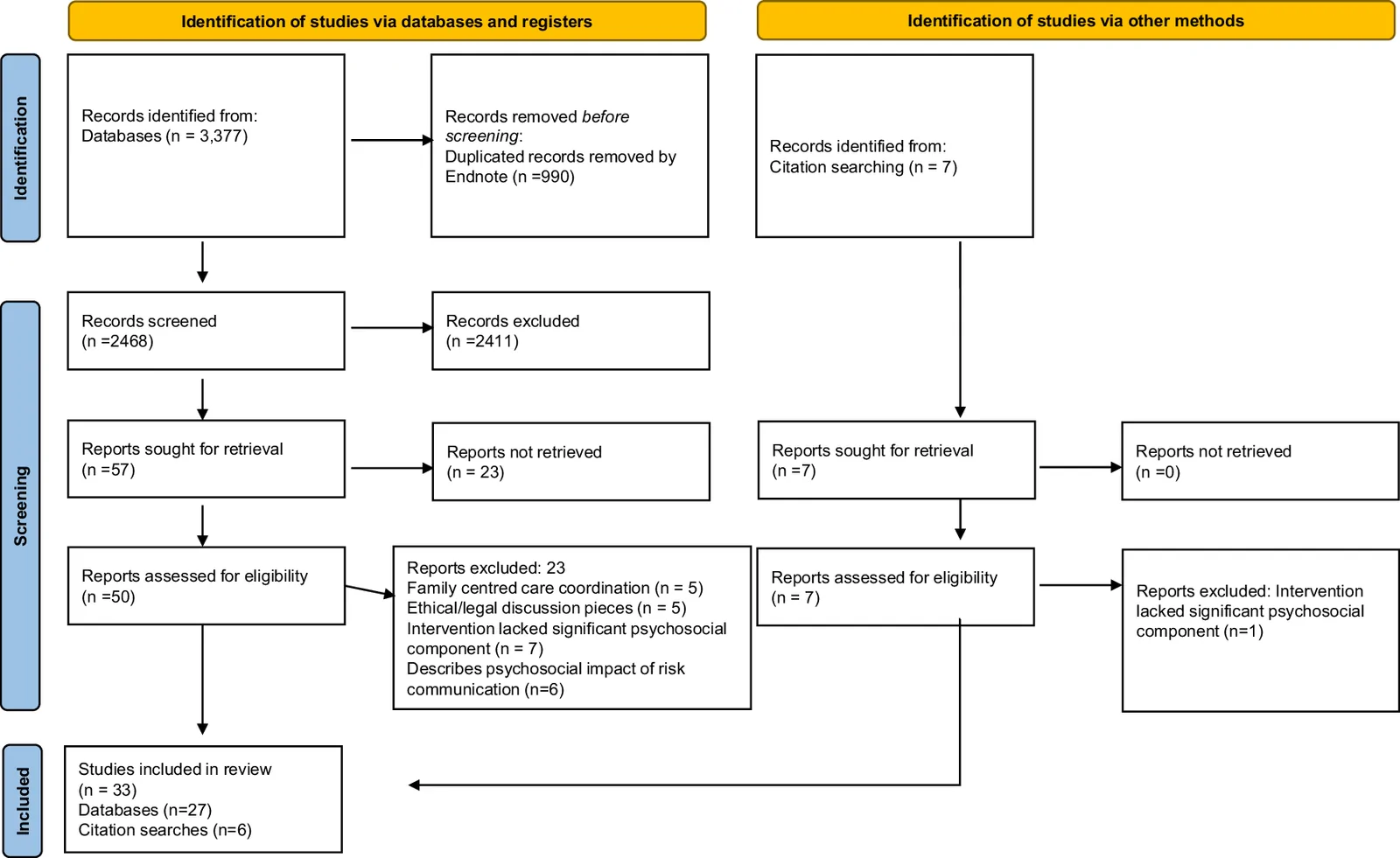
PRISMA-ScR flow diagram showing identification, screening and inclusion of publications. This figure summarises records identified through database searching and citation searching, duplicates removed, records screened, full-text reports assessed for eligibility, reasons for exclusion, and final publications included in the scoping review.

Eligibility criteria were defined using the PCC framework and are summarised in Table 1. We included empirical and non-empirical sources describing clinical practice or interventions with a clear ‘family element’ in the context of IGCs and excluded non-genetic contexts and individual-only interventions without a substantive psychosocial or family-systems component. Two reviewers (CG, HP) independently screened titles/abstracts and full texts; disagreements were resolved through discussion. Of 3377 records identified, 57 full texts were assessed and 33 publications were included. The selection process is shown in the PRISMA flow diagram (Fig. 1).

**Table 1:** Screening Strategy: Eligibility Criteria.

Domain	Inclusion	Exclusion
Population	• People with, or at risk of, an inherited genetic condition (IGC) • Relatives of affected individuals • Health professionals reporting experiences or views of interventions or practice with a ‘family element’	• Non-genetic conditions • Populations unrelated to genetic counselling contexts
Concept	• Clinical practice with a *family element* • Interventions aligned with family systems theory^1^ (e.g., family therapy, systemic therapy, dyadic approaches) • Discrete or practice-based interventions with family element and psychosocial components	• Individual-only interventions (e.g., CBT, skills training) • A discrete intervention that lacked a significant psychosocial component—e.g. pedigree drawing as sole family element; interventions where the primary outcome was uptake of genetic testing • Peer-only interventions without elements aligned with family systems theory. • Studies describing psychosocial issues without an intervention • Family centred care coordination*
Context	• Genetic counselling practice or settings involving support for families with inherited genetic conditions	• Non-genetic settings • Ethical/legal commentary • Clinical summaries or basic science reports
Types of Sources	• Empirical studies (qualitative, quantitative, mixed methods) • Commentaries, editorials, case studies, theses, conference abstracts	• Non-English publications • Review articles (though screened for relevant intervention references)

^1^ Interventions aligned with Family Systems Theory (e.g., family therapy, systemic therapy, dyadic approaches) are psychosocial approaches that view the family as an interdependent system and aim to improve communication, relationships, and adaptation by addressing relational patterns rather than individual symptoms (Bowen, 1978; Goldenberg & Goldenberg, 2013; MacLeod et al., 2021).

^2^Family-centred care coordination primarily addresses the organisation, delivery, and integration of healthcare services across providers and systems. In contrast, the review is concerned with supportive, communicative, and therapeutic interventions that enhance family relationships, adaptation, and communication in the context of genetic risk.

#### Data charting and synthesis

Data were charted using a JBI-adapted extraction form to capture study characteristics, clinical context, intervention features and reported outcomes, alongside fields reflecting key FSI/FSGI dimensions (illness trajectory, family-system variables, psychosocial context). Summary characteristics of all included publications are presented in Tables 2–4, with detailed intervention characteristics for the psychotherapeutic studies provided in Supplementary Material 3. We did not undertake formal critical appraisal, consistent with scoping review methodology.

**Table 2:** Summary of included publications and their position on the FSI/FSGI-derived family-oriented care continuum (Stages 1–3).

Category	Author/year	Design/type	Family element/focus	What it tells us about current practice	FSGI continuum: stage & gaps
**A. Clinical practice with a ‘family element’—original research (***n* = **12)**					
A	Clarke et al. [22]	Prospective multicentre study (*n* = 65 nondisclosure cases)	Nondisclosure of genetic risk within UK/AUS families	Nondisclosure rare but relationally driven (protection, conflict, estrangement). Clinicians encourage disclosure (follow-up, letters) but do not contact relatives without consent, highlighting tension between confidentiality and perceived duty to warn.	**Stage 1 → with Stage 2 awareness**. Stage 1 dominates: proband-centred, informational and ethically framed around individual. Stage 2 elements: explicit recognition of relational motives and emotional readiness. **Stage 3 absent**—no conjoint family work or longitudinal systemic follow-up.
A	Featherstone et al. [23]	Ethnography (62 consults, 22 home visits, 19 interviews, 35 meetings)	Kinship and family communication in genetics clinics	Shows how ‘family’ is actively constructed through pedigrees and risk talk; counselling is inherently relational and embedded in obligations and narratives. However, activity is organised around cases/probands and testing events.	**Stage 2 (hybrid)**. Rich systemic analysis but **practice remains largely Stage 1/2**: relational talk in service of risk communication. **Stage 3 structures (family as unit of care, planned family pathways) not present**.
A	Forrest et al. [24]	International GHCP survey (*n* = 626)	GHCP practices in communicating risk in families	93% identify at-risk relatives; 97% provide contact details; 59% offer follow-up, usually on patient request. Family letters and informational resources are standard, but emotional/relational support is ad hoc and time-limited.	**Stage 1 (proband-centred)**. Focus on illness trajectory and information transfer; family treated mainly as conduit for risk. **Stage 2 minimal (occasional exploration of family issues); Stage 3 absent**.
A	Gallo et al. [28]	Qualitative interviews (HCPs, *n* = 37)	Sharing information with families of children with a genetic condition	HCPs adapt explanations to family needs, age and parental preferences, but often defer decisions about telling children to parents. Emphasis on ‘getting the information right’ and avoiding harm.	**Stage 1 → modest Stage 2** features. Slightly more developmental/parenting focus than Forrest, but still predominantly biomedical and parent-centred. Systemic work with whole family or over time is not described **(Stage 3 absent**).
A	Mendes et al. [33]	Qualitative—GHCP views (*n* = 30)	Perceptions of a proposed multifamily intervention (MFDG) for cancer-risk families	HCPs see psychosocial and relational value in MFDGs but worry about time, remit, skills and funding. Family-oriented work framed as desirable but ‘remote possibility’.	**Stage 2 (aspiring to Stage 3)**. Conceptual endorsement of systemic care with services **operating at Stage 1–2**. Highlights implementation gap between values and available structures/skills.
A	Gaff & Hodgson [27]	Observational/training evaluation (*n* = 12 GCs)	GC training in a family-communication intervention	After training, >50% of GCs shift towards solution-focused conversations addressing broader family worries and communication tasks within standard appointments.	**Stage 2 (enhanced hybrid)**. Training deepens systemic questioning and planning but within **existing Stage-1 service constraints** (short, proband-centred consults)**. No transformation to Stage-3 pathways**.
A	Derbez et al. [30]	Ethnography (93 appts, 19 MDTs)	Support for disclosure in cancer genetics	Family communication addressed through pre- and post-test structures (MDTs, letters, follow-up). Clinicians sometimes revisit disclosure over time, especially in BRCA contexts.	**Stage 2 (structured hybrid)**. Integrates some family-system planning into the pathway, but relatives still approached via proband; psychosocial context (culture, stigma, SES) not systematically worked with. **Stage 3 absent**.
A	Eisler et al. [25]	Qualitative—GC interviews (*n* = 9)	GCs’ experiences delivering MFDGs after training	GCs endorse family-centred group work but feel under-prepared and squeezed by workload when asked to lead groups alongside usual duties.	**Stage 2 services + Stage 3 pilots**. MFDGs themselves are Stage-3 interventions, but **sit on top of Stage-2/1 services**. Lack of training/time prevents embedding systemic work as routine care.
A	Forbes Shepherd et al. [26]	GC interviews (n = 16)	Relational approach to HBOC counselling	GCs describe covert and overt strategies to navigate alliances, conflict, guilt and secrecy; relational negotiation is central, but organised through the proband’s consultation.	**High Stage 2**. Sophisticated systemic thinking, but action flows through individual appointments. **Stage 3 constrained** by remit, time and lack of structured family formats.
A	Keenan et al. [29]	Qualitative (GHCPs *n* = 23)	Facilitating parent–child communication in clinic	GHCPs vary widely in confidence and practice. Some revisit disclosure process across time; others avoid due to time, remit or perceived lack of skills.	**Stage 2 (patchy)**. Clear engagement with family-system variables (parent–child relationship, development), but support is informal, inconsistent, and proband-triggered. **Stage 3 not institutionalised**.
A	Young et al. [32]	Mixed-methods (surveys + interviews; GHCPs *n* = 73)	Challenges/strategies for supporting family communication	Strategies include early assessment of family dynamics, separate appointments, family letters, and psychosocial referrals. However, support is often reactive, dependent on patient request, and limited by time and remit.	**Stage 1** → **2 hybrid**. Stage 1 dominates: proband-centred, letter-based, and informational. **Stage 2 elements present:** some exploration of dynamics and emotional barriers. **Stage 3 absent:** no structured family-unit follow-up.
A	Mendes et al. [31]	Qualitative (GHCPs *n* = 34)	Professional roles in disclosure for untreatable conditions	GHCPs primarily see themselves as informing the proband, not disclosing to relatives; confidentiality prioritised, especially where no treatment exists. Support to wider family limited.	**Stage 1 (bio-ethical focus)**. Illness characteristics and ethical concerns dominate. Some relational reflection, but support remains firmly centred on individual autonomy, not systemic adaptation (Stage 3).
**B. Psychotherapeutic interventions with a ‘family element’—empirical evaluation (n** = **10)**					
B	Daly et al. [34]	Feasibility study (*n* = 38)	Genogram in cancer-risk counselling	Genogram found reliable and useful for exploring family relationships and patterns in cancer-risk consultations; prompts emotional and relational discussion.	**Stage 2 (assessment-focused)**. Brings family-system variables visibly into the room, beyond pedigree. Mainly a one-off mapping tool; not consistently linked to ongoing systemic intervention (Stage 3).
B	Peters et al. [35]	Feasibility (*n* = 20)	CEGRM—coloured eco-genetic relationship map	100% completion; supports mutuality, psychosocial insight and emotional expression for women with HBOC risk.	**Stage 2 → Stage 3 bridge**. Captures all FSGI domains (illness, relationships, context). In practice, mostly used as enhanced assessment; follow-on family work is optional or unspecified. Otherwise, **Stage-1/2 services**, rather than embedded in a systemic care pathway.
B	Peters et al. [36]	Larger feasibility/acceptability study (*n* = 150)	Adapted CEGRM in HBOC families	Highly acceptable; encourages sharing of emotions, social support and unresolved feelings.	**Stage 2 → Stage 3 bridge**. Demonstrates scalability and acceptability; however, CEGRM remains an adjunct tool within otherwise **Stage-1/2 services**, rather than embedded in a systemic care pathway.
B	Esplen et al. [38]	Phase II trial (*n* = 70)	Supportive-expressive group for BRCA1/2 carriers (inc. family session)	Group therapy reduced anxiety/depression; high satisfaction. Family session valued but attendance limited by time/distance; most participants attended alone.	**Strong Stage 2, partial Stage 3**. Intensive psychosocial support around illness and identity; family aspects acknowledged but relatives often absent. Systemic care is offered but not fully realised for whole family units.
B	McKinnon et al. [39]	Mixed-methods (*n* = 34)	BRCA multi-family retreat + updates	Retreat improves family communication, screening behaviour and peer support; no increase in distress. Families appreciate intensive shared space.	**Stage 3 (project-based)**. Fully engages illness trajectory, family process and context across multiple families. Not routine care—resource-intensive, time-limited and delivered as a one-off programme.
B	Mendes et al. [40]	Qualitative evaluation (*n* = 9)	MFDG for BRCA-risk families	Participants report benefits from group sharing, coping strategies and presence of medical staff; some techniques experienced as vague.	**Stage 3 (pilot)**. Clear systemic focus on joint family experiences and meaning-making. Small sample and early stage; sits alongside, not within, standard clinic pathways.
B	Mendes et al. [41]	Mixed-methods (*n* = 19; 20% dropout)	MFDG for colorectal-cancer–risk families	Supports shared experiences, perceived control over health, and action planning; practical barriers lead to attrition.	**Stage 3 (pilot)**. Demonstrates acceptability and systemic benefits, but engagement challenges and lack of integration into routine services limit reach.
B	Eisler et al. [37]	Development/feasibility study (*n* = 18 families)	Parent–child MFDG to facilitate communication about IGCs	Families report improved bonding and communication; mixed-condition groups acceptable; 100% completion in those recruited.	**Stage 3 (feasibility)**. Strong family-system orientation (joint sessions, developmental focus). Still single-site, evaluated as add-on, not yet a standard follow-up option.
B	Stopford et al. [44]	Mixed-methods evaluation (*n* = 6)	Narrative group for people after HD predictive testing	Provides safe space to revisit testing experience, share stories and explore coping. Participants value peer support; anxiety/depression unchanged.	**Stage 2 → early Stage 3**. Deep engagement with illness meaning and some family themes; groups mainly for individuals, not whole families. Systemic but not family-unit based.
B	Spiers et al. [43]	IPA qualitative (*n* = 12)	Narrative group for HD expansion carriers	Peer support, validation and improved communication about HD. Most find group helpful; two do not.	**Stage 2 → early Stage 3**. Narrative work strongly addresses relational processes and identity, but relatives are typically absent. Systemic ideas applied to individuals in group format.
**C. Family-oriented interventions aligned with family systems approaches—commentaries, case reports, book chapter, supervision papers (***n* = **11)**					
C	Eunpu [50]	Commentary	Systemically based psychotherapeutic techniques in genetic counselling	Outlines techniques (ecomaps, reframing, circular questioning) to move beyond individual focus; argues GCs should upskill in family-centred psychotherapy.	**Stage 3 model (conceptual)**. Provides a roadmap for fully systemic care, but no empirical data on implementation in routine genetics services.
C	Tuttle [48]	Commentary + case illustration	Experiential family therapy in genetic counselling	Describes immersive, multisensory, playful methods to reduce communication barriers in families; illustrated with clinical examples.	**Stage 3 therapy framewo**rk. Shows what systemic therapy can look like with genetics-affected families, but evidence limited to individual cases; not a service-level model.
C	Brown et al. [45]	Case report	Contextual family therapy for HBOC family (loyalty, legacy, ledger)	One in-depth case demonstrating long-term systemic therapy around hereditary cancer risk.	**Stage 3 (single case)**. Illuminates transgenerational ethics and loyalty processes; not evidence of widespread practice.
C	Kim [47]	Case report	Systems approach to counselling a family with albinism	Uses social ecology lens to map interactions between family, culture and stigma; informs tailored counselling.	**Stage 3** in one family. Fully FSGI-aligned conceptualisation; again single-case evidence, not routine service description.
C	Diekmann-Tapon [46]	Case report	Object-relations family therapy with couple after recurrent trisomies	Long-term psychoanalytic work on grief, guilt and early relationship patterns in a genetics context.	**Stage 3 specialised therapy**. Demonstrates depth possible when families access dedicated psychotherapy; not embedded in standard genetics pathways.
C	Speice et al. [42]	Practice report + qualitative feedback	Family issues in a BRCA psychoeducational group	Participants explicitly request more family-inclusive practice (joint sessions, peer linking, involvement of family therapists, follow-up planning).	**Stage 2 → Stage 3 signal**. Intervention itself is mostly **Stage 2**, but feedback clearly identifies unmet demand for structured Stage-3 family services.
C	McDaniel [4]	Conceptual + vignettes	‘Psychotherapy of genetics’: medical family therapy in IGCs	Multiple vignettes (HD, BRCA, HNPCC) demonstrate how family therapists can address anger, guilt, secrecy and decision-making across generations.	**Stage 3 theoretical framework, pockets of practice**. Argues strongly for integrated family therapy within genetics, but examples are local and therapist-dependent, not systemic service design.
C	McAllister et al. [52]	Book chapter (educational)	Supporting family communication about genetic conditions	Summarises tools (genograms, narrative approaches, life-cycle, MFDGs) and offers practical guidance for GCs.	**Stage 3 toolbox for practitioners**. Collates systemic techniques; does not itself evaluate or document how widely they are implemented.
C	MacLeod et al. [51]	Service description + framework	Narrative, family-systems approach in a UK genetics service	Describes narrative interventions (including MFDGs) integrated into follow-up for HD and other IGCs at Manchester; ongoing groups and clinics.	**Stage 3 practice within a single centre**. Demonstrates that embedded systemic care is possible, but scope is geographically limited and resource-intensive; not yet standard across services.
C	Ferrer-Duch [49]	Reflective practice paper	Systemic and narrative approaches in genetics (therapy, collaboration, Rare-D podcasts)	Illustrates collaboration between family therapist and genetics teams; describes direct family therapy, narrative groups and creative dissemination (podcasts) that support lived-experience narratives.	**Stage 3 contributions (local)**. Real-world systemic practice both in clinic and community; not yet mainstreamed or widely scaled.
C	Ferrer-Duch et al. [53]	Supervision model + early evaluation	Narrative-informed group counselling supervision (outsider-witness) for GCs	Long-running group supervision structure; GCs report feeling ‘energised’, more connected, and describe integrating narrative ideas into their work.	**Stage 3 thinking via capacity-building**. Systemic and narrative principles are embedded in GC supervision, indirectly shifting everyday practice along the continuum. Formal evaluation of family-level impact is still needed.

**Table 3:** Clinical practice with a ‘family element’—original research articles.

Paper	‘Family element’	Target	Purpose of article	Methods	Key findings
Clarke et al. [22]	Clinical Practice—nondisclosure of genetic risk within families	Proband & relatives (adult children, siblings, partners)	To prospectively investigate the frequency, circumstances, and professional responses when clients attending genetic clinics do not disclose relevant genetic information to at-risk relatives.	Prospective study across 14 genetics services (UK/Australia); 65 nondisclosure cases ( < 1% of ~38,700 consultations).	Nondisclosure mainly to adult children (39), siblings/relatives (22), partners (4).
				*Note: This is a clinician-reported study of practice (not self-report of attitudes only)*.	Most frequent conditions: Huntington’s disease, translocations, hereditary cancers.
					Reasons: protection from distress, assumptions relatives could not cope or did not need to know, family conflict/estrangement, privacy.
					Professionals encouraged disclosure via follow-up and family letters; no disclosure without consent.
					Highlights tension between nondirectiveness/confidentiality and duty to promote family disclosure.
					Authors suggest greater emphasis on support for family communication across generations, rather than regulatory enforcement.
Featherstone et al. [23]	Clinical Practice—ethnographic analysis of family and kinship in genetic counselling	Proband & family network	To explore how kinship and family are constructed and negotiated in the context of genetic counselling, and how genetic risks/diagnoses are embedded in family networks.	Ethnography of a UK genetics service; 62 consultations, 22 family home visits, 19 interviews, 35 meetings observed.	Families and kinship are not fixed but accomplished through social practice, including recognition, obligations, and shared narratives.
				*Note: This is a sociological ethnography of clinical practice, not a clinician self-report study*	The genetics clinic acts as a site of kinship construction, particularly through pedigree mapping and risk communication.
					Counselling encounters are inherently relational, highlighting the need for support that acknowledges both biomedical and social understandings of family. Clinicians work relationally but without systematic training, They recognise complexity but lack tools to address it, Their practice is constrained by biomedical assumptions
Forrest et al. [24]	Clinical Practice—supporting family communication	Proband	To investigate the extent to which GHCPs integrate the familial implications of an IGC into their practice and prove support around communication	International survey of 626 GHCPs (402 GCs)	GHPs typically encourage consultands to communicate IGC to at-risk relatives.
					GHPs less likely to identify relatives or encourage communication when the condition is untreatable.
					FU support (e.g., appts, calls) offered by some (59% offer follow-up appointments), but proactive contact with relatives is rare.
					Post-consultation support often requires initiative from consultands/family.
					8% of GHPs believe FU support is not needed. 41% never write letters for at-risk relatives
					Barriers include lack of time, admin/clinical support, and legal/ethical concerns.
Gallo et al. [28]	Clinical Practice—supporting family communication	Proband Parent	Examine HCPs views and strategies for individualising information sharing when working with families who have a child with a genetic condition	Interviews with 37 HCPs including 11 GHCPs (8 GCs and 3 CGs)	HCPs view parents as responsible for sharing genetic info with family.
					Emphasis on trusting, long-term relationships to support communication.
					Boundaries respected—professionals avoid direct contact with relatives.
					Support is mostly indirect (e.g., encouragement, written materials).
					Barriers include time, lack of systems, and unclear policies.
					Acknowledgement of family complexity and cultural considerations.
					GHCPS took responsibility for discussing familial implications.
Mendes et al. [33]	GHCPs views on Integration of MFDG intervention into clinical practice	Proband	To examine GHCPs opinions on MFDGs	3 interviews and 6 focus groups with 30 GHCPs (6 GC trainees)	Vast majority shared positive views on potential benefits to wellbeing and psychosocial support.
					Highlighted constraints: organisational structure, time, funding and skills.
					Overall, identified a gap between psychosocial need and service capacity.
Gaff et al. [27]	GCs delivery of intervention - supporting family communication	Proband	To design and implement an intervention to facilitate family communication	Observation of the training of 12 GCs	Over half GCs broke away from ‘client focused’ or ‘patient centred’ approach stipulated by the intervention design and employed ‘solution focused’ techniques which took in to account the implications for family members.
Derbez et al. [30]	Clinical Practice—supporting family communication	Proband	To identify key times when disclosure of genetic risk within family was addressed	Ethnographic study of (42 pretest & 51 result) appointments and MDT (19) clinics over 8 months	Family element is central throughout the genetic testing process.
					Pre-test: Disclosure is anticipated through family history questionnaires, pedigree mapping, and consent forms that address communication with relatives.
					Post-test: Healthcare providers emphasise the implications for family members, provide written materials to support disclosure, and offer psychosocial follow-up.
					Support strategies include: Framing disclosure as a family responsibility, supplying documents for relatives, offering direct or indirect disclosure (in line with French law), revisiting the issue during annual follow-ups, and involving community clinicians as additional points of contact
Eisler et al. [25]	GCs experience of delivering MFDGs	Proband	Explore whether GCs could deliver a MFDG and their views on	Interviews of 3 GCs	GCs perceived benefits of MFDGs for families and were positive about their experience. They were enthused by the opportunity to shift the focus of care from individualised to family centred care. However, they felt unconfident to deliver sessions.
					They saw potential to integrate into practice but perceived challenges of embedding it into a publicly funded health care system (funding, evidence about effectiveness, training requirements, human resources).
					GCs can be successfully trained to co-facilitate MFDGs, gaining confidence and therapeutic skills through hands-on practice and co-facilitation.
Forbes Shepherd et al. [26]	Clinical Practice—supporting family communication	Proband	To explore whether GCs use a relational approach to encourage genetic information disclosure	Interviews with 16 GCs	GCs employed relational approach in 3 escalating scales: covert, overt and authoritative. All approaches sought to make use of family-client relationships. Covert was most implemented. Overt approaches only used when disclosure issues became apparent. Authoritative approaches very rare and used specifically in cases of nondisclosure.
					Pedigree and consent forms used to incorporate familial elements.
					After results, GCs encouraged patients to get comfortable with diagnosis themselves and then disseminate at later date.
Keenan et al. [29]	Clinical Practice—supporting family communication	Proband	To gather the views and experiences of GHCPs about how they facilitate parent/child communication about IGCs.	Interviews with 23 GHCPs (10 GCs)	Only 7 participants felt confident supporting parent–child communication, with some offering direct support or speaking to children themselves.
					Others used follow-up (‘chipping away’) to encourage disclosure over time.
					Many viewed disclosure as the parent’s responsibility, offering only general advice, especially when unfamiliar with the child.
					Common challenges included uncertainty about timing and content of disclosure, differences in children’s maturity, and balancing parental autonomy with the child’s right to know.
Young et al. [32]	Clinical Practice—supporting family communication	Proband	To identify the most common challenges faced by GHPs when addressing family communication and explore strategies to manages these	Surveys and interviews with 73 GHCPs (59 GCs)	Key challenges: limited patient understanding of family communication importance, emotional barriers (guilt, anxiety, blame).Family conflict and estrangement (systemic issues common).
					Strategies: Early assessment of family dynamics, use of family letters and separate appointments, referral to psychosocial support when needed.
					Training needs: more support in reflective practice, behaviour change, and family therapy, uncertainty about depth of exploration in time-limited consults, peer debriefing valued; newer staff had more formal training.
Mendes et al. [31]	Clinical Practice—supporting family communication	Proband	Investigate how GHCPs perceive their roles and responsibilities when assisting patients with family disclosure	8 focus groups and 2 interviews with 34 GHCPS (1 GC)	GHCPs viewed their primary responsibility as informing patients about genetic risks to relatives, with patients holding responsibility for sharing this information.
					Most emphasised the importance of maintaining patient confidentiality, though some acknowledged a role in facilitating communication—particularly for actionable conditions.
					Less emphasis was placed on disclosure for untreatable conditions.
					GHCPs offered more support when patients raised communication difficulties, but time and resource constraints limited their capacity to address family communication consistently.

*GHCP(s)* Genetic Healthcare Professional(s), includes genetic counsellors, clinical geneticists, nurses, and psychologists working in genetic services, *GC(s)* Genetic Counsellor(s), *CG(s)* Clinical Geneticist(s), doctors who specialize in diagnosing and managing genetic disorders, *IGC(s)* Inherited Genetic Condition(s), genetic disorders passed from parents to children, *HCP(s)* Healthcare Professional(s), any professional working in a clinical healthcare setting, *MFDG(s)* Multi-Family Discussion Group(s), group sessions with several families to support communication and coping with genetic conditions, *FU support* Follow-up support, ongoing support such as follow-up appointments, phone calls, or further counselling after the initial consultation, *MDT clinic* Multidisciplinary Team clinic, a clinic where professionals from different medical specialties work together to support patients.

**Table 4:** Psychotherapeutic interventions with a ‘family element’—evaluation studies.

Author and year	‘Family element’	Target of Intervention	Purpose of article	Study methods	Key findings
*Family tree drawing techniques*					
Daly et al. [34]	GENOGRAM	Proband	Evaluate and validate the use of a genogram in context of cancer risk counselling technique	Drawing of genogram validated by comparing genogram to SASS.	· Two sources (genogram and SASS) were consistent.
				Sample: 38 females	
Peters et al. [35]	Colored, Eco-Genetic Relationship Map (CEGRM)	Proband	Evaluate the feasibility and utility of a coloured eco-genetic relationship map (CEGRM)	Compliance measured by % of participants who agreed to attempt intervention. Feasibility assessed by Likert scale and time to completion. Utility measured by additional psychosocial information gained.	· CEGRM construction was efficient with full participant compliance.
				Sample: 20 females	· Visualizing relationship patterns helped participants gain insights into their social networks.
					· Co-constructing the CEGRM encouraged active, mutual engagement and empathy.
					· Participants felt comfortable sharing meaningful stories about friends and family
Peters et al. [36]	Colored, Eco-Genetic Relationship Map (CEGRM)	Proband	Evaluate the feasibility and acceptability of an adapted coloured eco-genetic relationship map (CEGRM)	Compliance & feasibility assessed as per Peters et al. 2004.	· Compliance: 100% completion.
				Inferences about utility obtained by observation & open-ended responses during construction of CEGRM	· Feasibility: Scores averaged ~1.5, indicating ease of use.
				Sample:150 females	· Utility: Provided valuable psychosocial insights.
					· Empathic approach: Encouraged disclosure of deep, private emotions.
*Group therapy*					
Esplen et al. [38]	Supportive Expressive Group (inc family session)	Affected/at risk	To evaluate whether a group can help women cope with their cancer risk status.	Pre and post questionnaires (IES, BSI, TRIG, QLI and ad hoc questionnaires)	· Significant reductions in cancer worry, anxiety, and depression; other health-related and knowledge outcomes showed no statistical change.
				Content analysis for open-ended questions regarding potential additional benefits and satisfaction.	· High satisfaction (97%), with group support seen as beneficial.
				Sample: 70 women	· 72% valued help with family communication.
				(67 completed)	· Of 165 invited, 75 declined: 28% due to time constraints, 24% uninterested in group therapy, 21% felt location was too far despite living within an hour.
McKinnon et al. [39]	Multifamily discussion group (MFDG)	At risk/affected and 4 x partners	Evaluate a retreat for individuals and families with BRCA mutations	Pre and post session (6 months) = questionnaires (open and closed). IES and evaluation of the retreat.	· 50% of participants changed screening or prevention behaviours.
				Sample: 34	· 80% would attend a similar event again.
				4 males 30 females	· No change in psychological distress, but family communication improved.
					· Key benefits: connecting with others, shared family experience, access to information and psychological support.
					· Comfortable atmosphere (location, food) contributed to positive experience.
Mendes et al. [40]	Multifamily discussion group (MFDG)	At risk/affected	To evaluate a multi-family group intervention for women with BRCA mutations in the context of genetic counselling	Semi-structured focus group (90 mins) one month after the last session.	· Functional aspects (session number, duration) were adequate.
				Sample:	· Multifamily discussions were highly valued for promoting shared experience, reducing isolation, and offering practical coping strategies.
				9 (3 families)	· Doctor’s presence in session two was helpful for addressing medical questions.
				1 male 8 females	· Two participants found relaxation techniques vague and lacking structure.
					· Dropout rate was 25% (one family).
Mendes et al. [41]	Multifamily discussion group (MFDG)	At risk/affected	To evaluate a multi-family group intervention for at-risk people at risk of CRC in the context of genetic counselling	Semi-structured focus group (120 mins) one month after the last session.	· Practice aspects were adequate.
				Sample:	· MFDG format was a key benefit, fostering a supportive atmosphere and shared experiences.
				19 (4 families)	· Helped both newly and long-term at-risk individuals express emotions and serve as role models.
				6 males 13 females	· Educational session promoted a sense of control over health management.
					· Facilitators were seen as helpful.
					· Dropout rate was 20% (one family).
Eisler et al. [37]	Multifamily discussion group (MFDG)	At risk/ affected, including children and ‘not at risk’ family members	To ascertain whether an MFDG was a suitable approach and acceptable to both families affected by IGCs and GCs	Field notes and diary reflections (2x researchers)	· Families reportedly found the MFDGs group experience was highly beneficial in facilitating bonding and extrafamilial networks and improving psychological well-being.
				Thematic analysis of qualitative outcome measures, feedback and evaluation data and observational notes.	· Intervention structure and content acceptable - highlighted benefits of the facilitator’s role.
				Sample:	· Inclusion of families with different IGCs is valued.
				18 (6 families)	· Perception that it facilitated parent-child communication.
				5 males 13 females	· Compliance was 100%
Stopford et al. [44]	Narrative Therapy Groups (NT)	At risk	To ascertain the value and feasibility of employing narrative group sessions within the context of genetic counselling	In session observations. Written feedback forms (open and closed) at the end of the session.	· Responses were overwhelmingly positive.
		2 x partners (not at risk)		Semi- structured interviews after 6–8 week period.	· For many, it was the first time speaking openly about HD.
				GAD7 & PHQ9 pre- session and two weeks afterward.	· Anxiety levels remained low pre- and post-session.
				Sample :6	· GAD-7 and PHQ-9 were not statistically analysed due to a small sample; no clear effect was observed.
				3 males 3 females	· Themes: Session experience—safe, welcoming space; value of structure; benefit of group comparison and activities. Post-session reflections—feelings of connection, reassurance, and inspiration.
Spiers et al. [43]	Narrative Therapy Groups (NT)	At risk	Evaluation of participants' experiences of attending narrative therapy sessions to determine whether participating in a single genetic counselling narrative group is perceived as helpful	IPA interviews by an independent researcher. 2-–4 weeks after sessions.	· Most participants reported a very positive experience.
				Sample: 12	· Four themes emerged: The power of the group, Active elements of the narrative exercise, Subsequent impact, and a minor theme, Another Voice.
				2 males 10 females	· 11 of 12 participants valued connecting with others affected by HD and appreciated the peer support.
					· Empathic bonds formed with others living with the HD gene fault.
					· Participants felt more able to talk about HD with family and friends afterward.
					· Two participants found the session less helpful

*BSI* Brief Symptom Inventory, a self-report scale assessing emotional distress and psychiatric symptoms, *CEGRM* Colored Eco-Genetic Relationship Map, a visual tool used in genetic counselling to map social and family relationships and promote psychosocial reflection, *CRC* Colorectal Cancer, *GAD7* Generalized Anxiety Disorder-7, a 7-item questionnaire for assessing anxiety severity, *GC* Geneti c Counsellor, a professional who supports individuals and families in understanding and adapting to inherited conditions, ***HD*** Huntington’s Disease, a progressive genetic neurological disorder discussed in the context of narrative therapy, *IES* Impact of Event Scale, a tool to assess distress related to traumatic events, *IGC* Inherited Genetic Condition, a condition passed through generations by genes, central to many of the interventions reviewed, *IPA* Interpretative Phenomenological Analysis, a qualitative method exploring how people make sense of their experiences; *MFDG* Multifamily Discussion Group, a structured group intervention for multiple families facing similar genetic risks, *NT* Narrative Therapy, a therapeutic approach using storytelling to explore identity and experience in the context of illness, *PHQ9* Patient Health Questionnaire-9, a 9-item self-report measure used to assess depression severity, *QLI* Quality of Life Index, a tool for measuring overall well-being and life satisfaction, *SASS* Social Adjustment Scale Self-Report, a measure of social functioning across domains such as work, family, and social life, *ToL* Tree of Life, a narrative therapy metaphor and visual technique used to explore personal strengths, family stories, and coping.

Charted data were synthesised narratively, organised initially around our three a priori domains: (1) conceptualisation, (2) implementation and (3) evaluation of family-oriented support. We used Rolland’s FSI/FSGI frameworks as sensitising frameworks to guide data charting and narrative synthesis, rather than as formal scoring tools. This meant that publications were not categorised as family-oriented simply because they referred to relatives or family communication. Instead, we examined how far each paper engaged with the interaction between genetic risk, family relationships and wider psychosocial context. For each publication, we considered four dimensions: (1) illness/risk trajectory, including the timing of testing or diagnosis, predictability, actionability, chronicity, reproductive implications and whether support extended beyond the initial testing event; (2) family-system processes, including communication patterns, secrecy or openness, conflict, guilt, roles, boundaries, couple and parent–child relationships, intergenerational meanings and family decision-making; (3) psychosocial context, including emotional readiness, prior family illness experiences, stigma, cultural or religious beliefs, socioeconomic and geographical barriers, available social support and the capacity of services to provide follow-up; and (4) intervention structure, including who was involved, whether relatives were directly included, whether the family was treated as a unit of care, the timing and intensity of support, and whether support was reactive, optional or built into the care pathway. These dimensions informed the development of a three-stage heuristic continuum of family-oriented care. Publications were positioned toward Stage 1 where family work was mainly informational, proband-mediated and focused on disclosure or risk transmission; toward Stage 2 where clinicians assessed or responded to family dynamics and psychosocial barriers but within individualised or ad hoc service structures; and toward Stage 3 where interventions actively worked with family members or multiple families as the unit of care, using systemic, narrative or psychotherapeutic methods to support communication, adaptation and shared meaning-making over time. For descriptive purposes, we refer to empirical studies of routine practice as ‘group A’, evaluated psychotherapeutic interventions as ‘group B’, and conceptual or descriptive psychotherapeutic papers as ‘group C’. Further detail on the search strategy and the application of the FSI/FSGI-informed analytic dimensions, including examples of how publications were positioned across the continuum, is provided in Supplementary Material 1.

## Results

Thirty-three publications were included: ten conceptual or descriptive papers, 22 empirical studies, and one book chapter. Findings were grouped into two categories: (1) descriptions of clinical practice with a ‘family element’ (Group A), and (2) psychotherapeutic approaches to supporting people with IGCs that included a ‘family element’ (Group B and C). Twelve studies reported on routine practice, mainly exploring how GHCPs guided family communication of risk (Group A). Twenty-one publications described psychotherapeutic approaches, ten of which were discrete interventions formally evaluated by study design (Group B and C). Tables 2–4 and Supplementary Material 3 provide an overview of included articles.

### Clinical practice with a ‘family element’

#### Group A

Twelve empirical studies investigated how family considerations are integrated into clinical genetics practice (Table 3). Early UK work included a multicentre survey on nondisclosure within families [22] and an ethnographic study of how kinship and responsibility were negotiated in the genetics clinic [23]. The remaining ten studies examined GHCPs’ practice in supporting family communication. One was a large multinational survey of 626 GHCPs [24]; the others used qualitative (*n* = 8) or mixed-methods (*n* = 1) designs. Participants included GCs, clinical geneticists, nurses, psychologists and other allied HCPs, with several studies focusing solely on GCs [25–27]. All were conducted in Western, high-income settings.

#### Practices and strategies

All practice studies explored how GHCPs encouraged dissemination of genetic risk information within families. Clarke et al. [22] found nondisclosure to be rare ( < 1% of ~38,700 consultations), usually involving parents protecting adult children; clinicians encouraged disclosure but did not breach consent, highlighting tensions between confidentiality and duty to warn. Featherstone et al. [23] showed how pedigrees and risk talk actively constructed kinship and responsibility, with counselling inherently relational.

Across later studies, GHCPs were generally proactive in sharing generic risk information. These strategies remained largely informational and proband-centred (Stage 1), with emerging but inconsistently applied Stage-2 elements (reflective questioning about family dynamics, occasional joint family appointments, ad hoc psychosocial referrals). In Forrest et al.’s survey, 93% reported always identifying at-risk relatives and encouraging intrafamilial communication [24], yet some clinicians perceived involvement in family communication as outside their remit [24, 28, 29], while others described more directive approaches, including offering direct contact with relatives [29–31]. Overall, authoritative strategies were rare; indirect prompts and gentle encouragement predominated [26, 29–32].

The pre-test consultation was widely seen as a key space for family-oriented work. Pedigrees and consent forms were used to visualise relationships, assess family dynamics and foreground familial implications [23, 26, 30, 32]. Some GHCPs used reflective skills to explore expected disclosure challenges and to cue patients to anticipate difficult conversations [26, 30, 32].

**Fig. 2:**
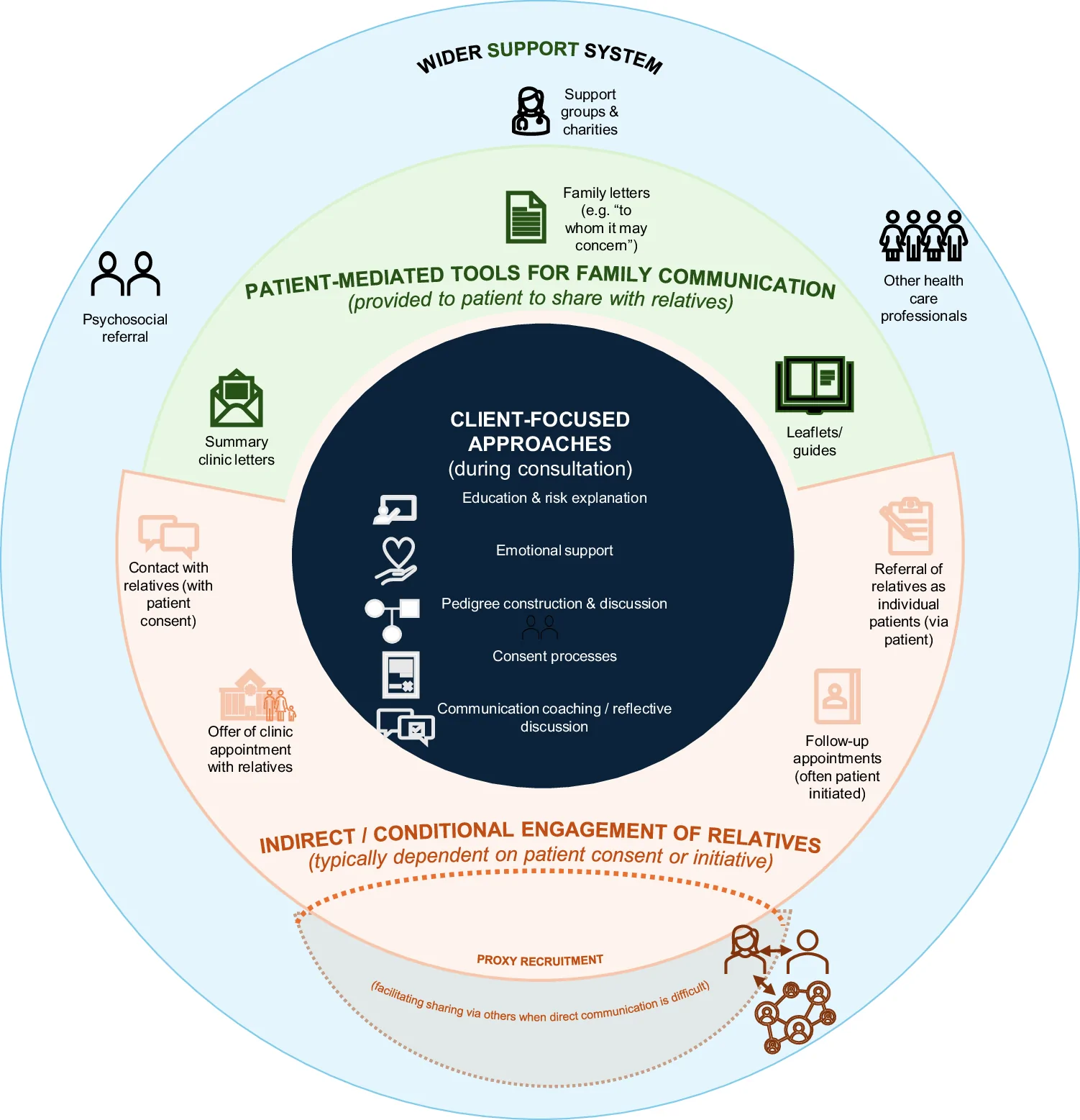
Summary of approaches and resources used to facilitate family communication. This diagram summarises strategies identified across included clinical practice studies. The inner circle represents client-focused approaches delivered within the consultation, including education, emotional support, and communication coaching. The middle layer shows patient-mediated tools provided to support communication with relatives, such as family letters, written materials, and summary clinic letters. The boundary layer reflects mechanisms through which relatives may be indirectly or conditionally engaged, typically dependent on patient consent or initiative, including offers of appointments or contact with relatives. The outer layer represents wider professional and community support systems, including referrals to other healthcare professionals, psychosocial services, and support organisations. Across all domains, strategies rely predominantly on the patient to initiate or enable communication with relatives. For example, ‘To Whom It May Concern’ letters must be physically or digitally shared by the patient (sometimes anonymously), and direct contact with relatives or onward referrals almost always require patient consent. This structure reflects the prevailing model of genetic counselling, in which the patient remains the primary conduit for disseminating genetic risk information within the family.

Post-test support varied. In Forrest et al. [24], most GHCPs reported offering contact details (97%) and follow-up appointments (59%), but several studies suggested that additional support was typically patient-initiated rather than systematic [24, 31, 32]. Clinicians were more likely to revisit disclosure when patients raised concerns [24, 30, 32]; some routinely checked on intrafamilial communication at follow-up [26, 30], but in-depth counselling techniques were rarely described. The most common follow-up support comprised informational prompts and written resources (e.g. family letters) to facilitate disclosure (summarised in Fig. 2).

Although information-giving dominated, some GHCPs explicitly recognised that patients needed to be emotionally ready to disclose [22, 28–30, 32]. A minority offered ongoing emotional support or tried to make joint family appointments more inclusive (e.g. attention to seating, ensuring all voices were heard) [32]. Across studies, GHCPs were more likely to invest in disclosure support when conditions were actionable and less likely when no medical management was available [22, 24, 31].

#### Barriers and limitations

Across studies, a consistent gap emerged between recognised psychosocial needs and the capacity of services to address them. From an FSI/FSGI perspective, most services operated firmly within Stage 1, with only patchy movement towards early Stage 2. Family-system variables (e.g. conflict, estrangement, guilt) were seen as relevant but rarely addressed through structured, longitudinal support. No study described a service in which the family was systematically treated as the unit of care (Stage 3).

Support was primarily targeted at the individual proband. Forrest et al. [24] reported that at-risk relatives could be offered clinic appointments (66%), but usually only once referred as individual patients. Many GHCPs understood their main responsibility as providing objective risk information, leaving intrafamilial communication to the proband [22, 24–26, 28, 31]. Tensions between autonomy, confidentiality and perceived duties to relatives were common [22, 26–29, 31, 32]. Where family consultations occurred, the focus remained on individual testing and decision-making [32], and balancing individual and family needs was described as one of the most challenging aspects of practice [26, 32].

Across studies, GHCPs expressed a sense of responsibility to support wider family members but variable confidence in how far to pursue relational work [28, 29, 31, 32]. Many reported feeling under-skilled or under-resourced to address complex family dynamics and emotional responses [25, 31, 32], often referring on to psychology when difficulties exceeded their perceived remit [29, 30, 32]. Training needs included reflective practice, behaviour-change strategies and family systems theory, yet even GCs with some training reported uneven confidence [32]. Time, workload and administrative barriers were frequently cited [24, 29, 31–33]; in one survey, 60% reported lack of time as limiting support [24].

#### Psychotherapeutic approaches with a ‘family element’

Twenty-one publications integrated family systems or psychotherapeutic principles into genetic counselling or closely related settings (Table 2; Supplementary Material 3). Together, they spanned both direct therapeutic interventions for families and indirect models designed to enhance family-oriented practice.

#### Group B

Within this set, ten papers reported formally evaluated interventions (Table 4; characteristics in Supplementary Material 3). These comprised three studies of in-session assessment tools—genogram-based exploration of family relationships [34] and two linked iterations of the coloured eco-genetic relationship map (CEGRM) [35, 36]—and seven structured group programmes. Group interventions included multifamily discussion groups (MFDGs), supportive–expressive and psychoeducational groups, retreat-based formats and narrative group sessions [37–44].

#### Group C

Eleven additional publications were conceptual or descriptive. Some presented case reports or service descriptions applying systemic or narrative family therapy with families affected by IGCs [45–49]. Others outlined broader models or educational and training frameworks for integrating family systems theory and techniques (e.g. genograms, family life-cycle work, multifamily formats) into practice, including one book chapter [4, 50–52]. Finally, two papers described narrative-informed supervision and service structures that indirectly support family-oriented practice by sustaining GCs’ relational capacity and embedding systemic thinking within services [51, 53].

#### Characteristics of evaluated interventions (Group B)

Of the ten intervention studies, three were UK-based [37, 43, 44], four from the USA [34–36, 39], two from Portugal [40, 41] and one from Australia/Canada [38]. Three extended pedigree methods into psychosocial mapping within individual sessions [34–36]. The remaining seven used group formats grounded in systemic or narrative approaches [37–41, 43, 44]; four incorporated structured biomedical education [38–41]. Most focused on adult-onset conditions: six in hereditary breast/ovarian cancer (HBOC) [34–36, 38–40], one in hereditary colorectal cancer [41] and two in Huntington’s disease (HD) [43, 44]. Eisler et al. [37] included mixed IGCs and children under 12.

Across studies, samples were predominantly female, white and well-educated, with limited reporting of socio-economic or cultural diversity. Only four interventions explicitly included family members not personally at risk [37–39, 44]. Group sizes ranged from six to 41; six interventions were single-session, and the longest (supportive-expressive therapy) spanned 12 sessions over six months [38]. All interventions were delivered face-to-face, usually within hospital-based genetics services, although one took place in a rural retreat setting [39]. Multidisciplinary facilitation was common; psychologists/family therapists often co-facilitated with GCs [37, 38, 43, 44]. Attendance and completion rates were generally high ( ≥ 75%, often 100%).

#### Acceptability, feasibility and psychological impact

Across studies, the primary aim was to assess feasibility and acceptability, with secondary attention to coping and communication outcomes. Participants tended to view the interventions positively [37–44]. Functional features such as structure, duration and format were largely acceptable; some participants requested longer sessions despite time demands [37, 41]. The most intensive programme reported a 45% refusal rate, often due to travel and childcare constraints [37, 38]. Hospital environments were sometimes experienced as less conducive to open discussion than non-clinical or retreat settings [37, 39, 44]. Group diversity was generally welcomed, although some participants preferred peers in similar life or adaptation stages [37, 44]. Facilitators with both psychosocial and genetic expertise were particularly valued [37, 40, 41], and blended educational–emotional content was consistently highlighted as helpful [37, 39–41].

Quantitative findings on psychological outcomes were mixed. One phase-II trial reported reductions in cancer-related worry, anxiety and depression following supportive-expressive group therapy [38]. Other studies noted stable distress scores despite positive qualitative feedback [39, 44]. Across interventions, qualitative accounts were more uniformly positive: participants described feeling less isolated, more confident, better able to share difficult emotions and equipped with new coping strategies [34–44].

#### Impact on family communication

Four studies explicitly reported communication outcomes [37–39, 43]. Participants described greater confidence or insight for talking with relatives [38, 43], and Eisler et al. [37] provided rich accounts of enhanced parent–child communication. However, most interventions were not powered to detect change, and quantitative measurement of communication was sparse. Several studies noted that many participants had already disclosed genetic risk prior to the intervention [37, 39], limiting the scope for observable change in disclosure behaviour.

#### The continuum of family-oriented care

**Fig. 3:**
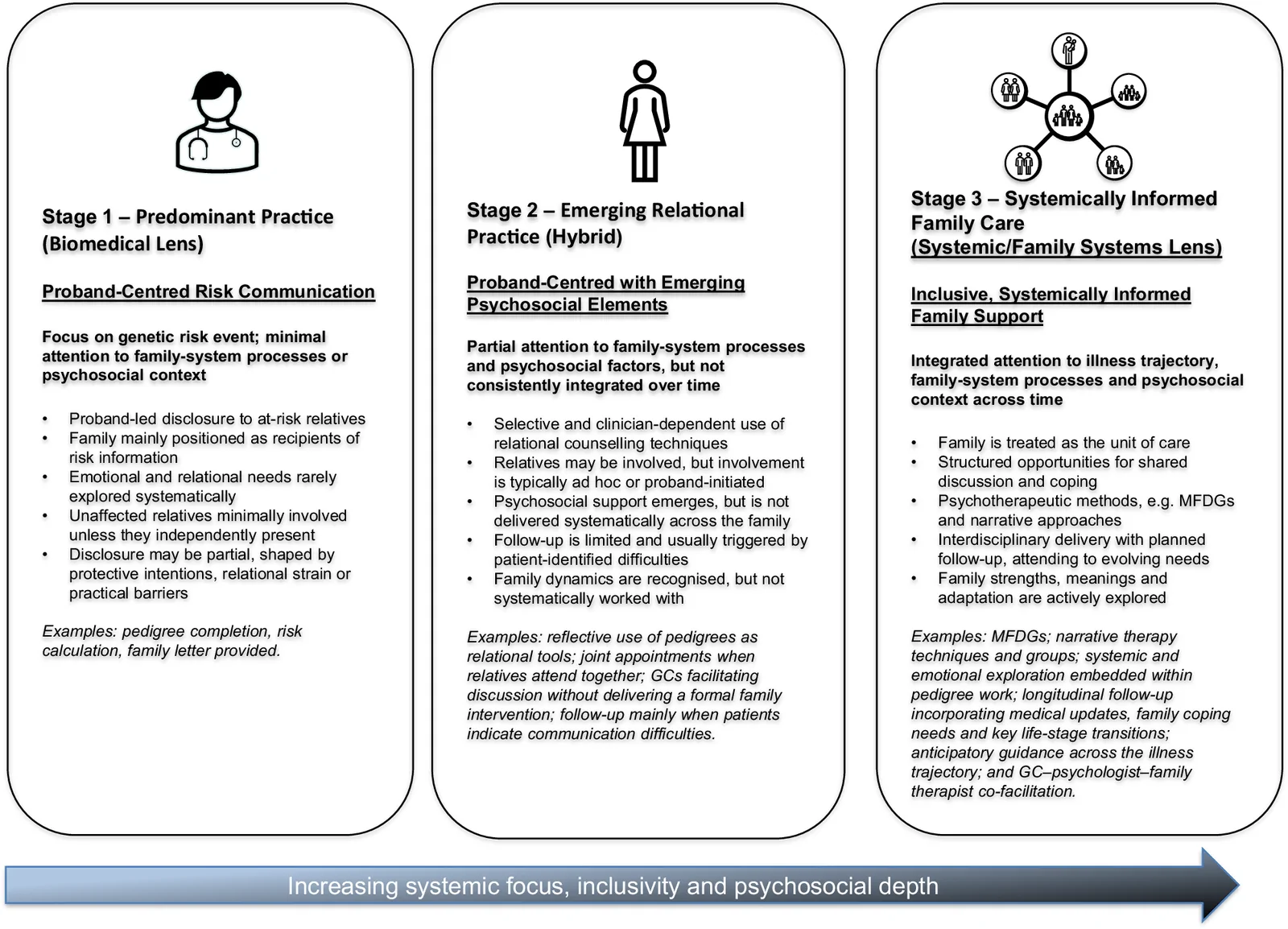
Continuum of family-oriented support in genetic counselling: from risk communication to systemic family care. This figure presents a conceptual and interpretive synthesis, informed by Rolland’s Family Systems Illness/Family Systems Genetic Illness frameworks, illustrating the progression from standard genetic counselling practices focused primarily on the individual to increasingly structured and systemic family-oriented interventions. Standard approaches include information provision, pedigree construction, risk assessment and encouragement of family communication. Intermediate approaches include relational techniques such as reflective pedigree use, joint consultations and discussion of family dynamics. At the most intensive level, interventions draw on family systems and psychotherapeutic models, treating the family as the unit of care. The continuum reflects variation in intensity, theoretical underpinning, psychosocial depth and degree of family involvement identified across included studies.

Drawing on the FSI/FSGI frameworks, we developed a three-stage continuum to locate the practices and interventions identified in this review. The continuum is intended as a heuristic representation of how fully current practices operationalise core FSI/FSGI principles, rather than as a validated classification tool (Fig. 3). Figure 3 should therefore be read alongside Supplementary Material 1, which provides additional detail on how the FSI/FSGI-informed analytic dimensions were applied interpretively to develop the continuum.

Stage 1—Biomedical, proband-centred: care focuses on identifying at-risk relatives and supporting the proband’s disclosure. The family is viewed mainly as a conduit for risk information, with minimal structured engagement of other members and limited attention to emotional or relational needs.

Stage 2—Hybrid: some psychosocial and relational elements are incorporated (e.g. reflective questioning, occasional joint family appointments, use of genograms/CEGRMs, group-based support), but involvement of wider family members is informal, optional and typically initiated by the proband.

Stage 3—Systemic family-oriented care: the family (those at risk and those emotionally affected) is conceptualised as the unit of care. Structured opportunities are created for shared discussion, adaptation and coping, delivered by interdisciplinary teams and tailored to illness trajectory, life stage and evolving needs.

Across publications, routine clinical practice aligned mainly with Stage 1, with pockets of Stage-2 activity. The ten evaluated interventions spanned enhanced assessment tools (Stage-2 → 3 bridge) and genuinely systemic group formats (Stage 3), but all were delivered as adjuncts to standard, predominantly Stage-1 services. Although MacLeod et al. [51] and Ferrer-Duch [49, 53] describe narrative and systemic approaches embedded within specific UK services, these remain local exemplars rather than widely adopted service models.

## Discussion

This scoping review mapped clinical strategies and psychotherapeutic interventions for families affected by IGCs to Rolland’s FSI/FSGI frameworks. Across the literature, most practice remained at the biomedical, proband-centred end of the continuum (Stage 1), with some movement toward hybrid psychosocial approaches (Stage 2). Psychotherapeutic interventions demonstrated feasibility and perceived benefit for participants but were typically adjuncts rather than embedded components of routine care. Fully systemic, family-level care (Stage 3) was not identified in the standard service models reported. From an FSI/FSGI perspective, this pattern is unsurprising: without explicit structural and temporal support for relational work, services tend to default to individualised, information-driven practice.

This continuum should therefore be read as an interpretive map of the literature, rather than as a formal rating system. Its purpose was to make visible a distinction that was otherwise blurred across studies: many publications recognised that genetic risk is familial, but fewer described care structures that actively engaged the family system. This was particularly important in relation to the psychosocial context. Emotional readiness, family beliefs, communication norms, stigma, cultural values, social support and service capacity were often acknowledged as relevant to disclosure and adaptation, but were not consistently assessed, revisited or therapeutically addressed. We therefore interpreted many studies as occupying a Stage 1–2 or Stage 2 position: the family was conceptually present, but not consistently present as the unit of care.

### Conceptualisation of family support

Across the publications included in this scoping review—and consistent with the wider literature tracing the evolution of genetic counselling practice [5–15], family-oriented support in empirical studies of routine practice (group A) was typically conceptualised narrowly, with the proband positioned as the primary conduit of genetic risk. GHCPs commonly encouraged patient-led disclosure, supported by letters or generic written resources, while wider relational and emotional needs were explored inconsistently [22–32]. On our FSI/FSGI-derived continuum, this maps largely to Stage 1 (biomedical, proband-centred care): the family is recognised as genetically relevant, but not consistently treated as a unit of care.

This stands in marked contrast to the conceptual and commentary literature (group C), which has, since the late 1990s, articulated rich systemic, narrative and developmental frameworks for working with families affected by IGCs [4, 45–53]. These papers explicitly describe the family as the unit of care and outline clear strategies for supporting communication, shared meaning-making and adaptation across the illness trajectory (e.g. intersystem formulations, contextual therapy, experiential and narrative approaches, MFDGs, life-cycle-informed work). From an FSI/FSGI perspective, these models are firmly aligned with Stage 3 systemic family-oriented care, even though they are rarely embedded in service-wide pathways.

At the same time, our synthesis suggests that systemic practice is already present in pockets, but tacit and inconsistent, cutting across both empirical intervention studies (group B) and some descriptions of routine care (group A). Many of the techniques identified in our review are *implicitly* systemic, but not always labelled as ‘family therapy’ or ‘systems-based’ by the authors. Supportive-expressive groups for BRCA carriers [38] and McKinnon et al.’s post-testing intervention [39], for example, were framed primarily in psychoeducational or support terms, yet worked with family narratives, roles and mutual support in ways that resonate strongly with FSI/FSGI principles.

Taken together, the picture is of a field with strong systemic conceptual foundations (group C) and growing but fragmented, sometimes unnamed systemic practice (groups A and B), consistent with findings from Dane et al. [54]. Rather than requiring a wholesale reinvention of genetic counselling, the challenge is to make these systemic elements explicit, name them, and support their consistent use—moving from isolated, condition-specific or research-only initiatives toward routinely available, Stage-3 systemic family-oriented care that is resourced, evaluated and integrated into mainstream pathways.

### Gaps in implementation and delivery

From a family systems perspective, the lack of longitudinal follow-up is striking. Rolland’s FSI/FSGI frameworks emphasise ‘psychosocial check-ups,’ in which HCPs periodically revisit salient issues and priorities as they surface and change across illness phases [19, 20]. In contrast, most practices and interventions described in this review were effectively one-off, tied to specific testing or diagnostic events, with little attention to difficulties that emerge over time. Embedding multiple, low-intensity touchpoints—via in-person, telehealth or digital modalities—could allow clinicians to identify shifting strengths and vulnerabilities in family functioning, aligned with illness phase and life-cycle stage.

All interventions were delivered face-to-face, usually within hospital-based genetic services. For families affected by rare IGCs, who are often widely dispersed, exclusive reliance on in-person formats limits accessibility. Virtual and hybrid formats—telehealth-based family sessions, online groups, or digitally delivered psychoeducational resources—offer opportunities to widen access and reduce travel burden, provided their design explicitly preserves the relational, interactive and systemic elements central to systemic family-oriented care. Importantly, most participants had already received genetic counselling, which itself (particularly when enhanced) is a complex intervention that can support family communication [55]. As such, these interventions may not have targeted families with the greatest unmet need.

#### Demographic gaps

The review highlighted significant demographic gaps in the studies and interventions included. Specifically, there was under-representation of certain populations, including those with untreatable or unpredictable conditions, men, partners, children, and non-Western groups. Several of these groups—notably men and Black women—have previously been identified as underserved and with unmet needs in genetic services [56–58]. Most interventions were concentrated in HBOC contexts, leaving conditions with less predictable or actionable outcomes largely unaddressed.

Studies predominantly involved participants from Western, high-income countries and largely focused on female, well-educated populations. There is a need for more inclusive research that captures the experiences of underrepresented relatives (e.g., unaffected family members) and diverse cultural groups.

### Mainstreaming genomics and the shrinking space for psychosocial work

The mainstreaming of genomic testing—here referring to the growing provision of genomic testing by non-genetics clinicians in specialties such as oncology, cardiology and neurology, rather than solely within specialist clinical genetics services—represents a critical inflection point for family-oriented care. Studies in this review found that pre-test genetic counselling delivered by GCs has been the main setting in which clinicians assess family dynamics, explore relational patterns, anticipate disclosure challenges and support emotional preparation for testing and its possible outcomes [23, 26, 30, 32]. Specialist clinical genetics services have long been attuned to the familial implications of genetic information, including its relevance for relatives as well as the patient. As testing is increasingly delivered in mainstream settings, however, attention may be focused more narrowly on the individual patient, with less space to anticipate and address the wider familial and relational implications of results [7, 12–14]. At the same time, our review and others indicate that post-test support remains inconsistent and largely patient-initiated, with few systematic mechanisms for revisiting intrafamilial communication or psychosocial adaptation over time [15, 24, 30, 32]. When relational preparation is diluted upstream and follow-up remains fragmented downstream, families may face the most challenging phases of adjustment with little structured support.

International consensus work echoes these concerns. Phillips et al. (2025) identified five priorities for addressing the familial implications of genomic findings: recognising family communication as an ongoing process, adopting family- rather than individual-centred models, clarifying roles and responsibilities, developing clear guidance and policy, and ensuring adequate resourcing [7]. Our synthesis reinforces these priorities and suggests that, without intentional redesign, mainstream genomics risks further entrenching a Stage 1, transactional model and eroding core counselling competencies—systemic assessment, emotional attunement and facilitation of shared meaning-making—that are central to family adaptation.

### Evaluation: what we measure and what we miss

Evaluation of family-oriented support remains limited and uneven. Most clinical practice studies relied on clinician self-report regarding strategies used, rather than direct observation or outcome data. Intervention studies primarily assessed feasibility and acceptability, with small sample sizes and limited follow-up. Qualitative feedback consistently indicated emotional and relational benefits (reduced isolation, increased confidence, improved communication), whereas quantitative findings were modest, mixed or null. Only a minority of evaluations measured family communication directly [37, 39].

Recent reviews echo these gaps. Zhao et al. [18] found that most family communication interventions in genetics were rooted in educational or behaviour-change models, with little attention to systemic evaluation. Barbosa et al. [59] highlighted the dominance of linear ‘sender–receiver’ frameworks in both conceptualisation and measurement. From an FSI/FSGI perspective, this reflects a misalignment between what is measured and what matters: Rolland emphasises family adaptation across time phases and contexts, yet most existing evaluations capture short-term disclosure behaviours or individual emotional states, overlooking relational processes and long-term systemic adaptation.

### Moving toward systemic family oriented care: implications for practice and policy

#### Building systemic capacity in clinical practice

A truly systemic family-oriented model must address the emotional and adaptive needs of the wider family system—not just the proband—recognising that psychological processes in families can influence patterns of engagement, health behaviour and clinical outcomes. Moving from Stage 1 (biomedical, proband-centred care) toward Stage 3 (systemic family care) will require both structural and skills-based change. Interdisciplinary delivery models, systemic supervision, and the integration of therapeutic formats—such as multifamily discussion groups, narrative approaches and strengths-based interventions—can help embed systemic thinking within routine pathways. The multifamily discussion groups, narrative HD groups and supportive–expressive interventions in this review [37–41, 43, 44] provide working prototypes of Stage-3 care that could be adapted and scaled, for example as routine follow-up offers post-testing. Even when not all families require or choose intensive interventions, having these options visible and normalised signals that families, not just individuals, are legitimate recipients of care.

#### Navigating ethical dimensions

Ethical tensions are inherent in family-oriented genetic care because genetic information is simultaneously personal and shared: respecting one person’s autonomy, privacy and preferences may at times sit uneasily alongside potential benefits to relatives who may also be affected by or at risk from the same information. Multiple studies in this review illustrated how confidentiality and nondisclosure create dilemmas for GHCPs around the limits of their involvement [22, 31, 32]. Family-centred approaches, while valuable, may inadvertently generate pressure to disclose or amplify guilt and blame. Clear ethical guidance, decision pathways and reflective practice structures are therefore essential to ensure that systemic models support—rather than compromise—autonomy, psychological safety and voluntary disclosure.

#### Genetic counselling workforce and digital tools

Workforce capacity is a major barrier to implementing systemic models, particularly as genomic testing expands. While some tasks can be shared with or delegated to other professionals, the distinctive contribution of GCs—integrating advanced genomic knowledge with specialised counselling skills—remains central. As genomics becomes further mainstreamed, it is vital that GCs retain protected time to develop and apply these psychosocial and systemic competencies rather than being pulled predominantly into technical or informational roles.

Digital tools, such as web-based modules and AI-supported resources, can help offload certain ‘teaching’ tasks in genetic counselling. However, any time saved should be dedicated to relational and systemic tasks. In outcome-driven services, efficiencies gained from these tools may simply be reinvested into increased throughput and information delivery. The real value of genetic counselling lies in relationship-building, counselling, and systemic support, which are harder to quantify [4, 9–11].

Recent emerging evidence suggests that some digital solutions for information delivery may not compensate for the loss of relational space. In a large study, Mahal et al. (2025) found that pre-consultation videos in hereditary cancer and syndromic genetics clinics were helpful but did not reduce clinician workload [60]. For more complex cases, these videos led to lower patient satisfaction and higher clinician burden, suggesting that informational front-loading may not suit situations with high uncertainty and relational complexity. Rather than freeing up time for psychosocial work, these tools may simply add more informational layers without addressing the structural constraints on relational practice.

Diversifying who delivers which elements of family-oriented care will be essential if services are to remain sustainable. Embedding psychologists within genetics services [61] or co-facilitating groups with systemic therapists [43, 44] can further enhance relational capacity and ensure that communication, coping and meaning-making are addressed across the illness trajectory. These approaches should complement, rather than replace, genetic counselling; GCs are well placed to co-design, co-facilitate and provide consultation to interdisciplinary and peer-supported programmes, ensuring that systemic principles and ethical safeguards are embedded from the outset.

From a health-economic perspective, investing in family-centred models is also rational. Working with families as interconnected systems, rather than as isolated probands, may enhance cascade testing, reduce duplication of consultations across relatives, and promote earlier surveillance or prevention—likely less costly than managing advanced disease or fragmented repeat contacts. In a constrained workforce, systemic family-oriented approaches may therefore maximise the impact of each encounter by leveraging one consultation to support multiple at-risk relatives, aligning good psychosocial care with more efficient resource use.

#### Extending systemic care beyond the clinic and across cultures

Systemic care must also extend beyond clinic walls. Families affected by inherited conditions navigate complex emotional, cultural and social landscapes that span multiple settings and life stages. Community-based and adjunctive strategies—such as podcasts, online communities, social media, and partnerships with trusted local organisations—can reinforce therapeutic work, reach individuals not engaged with specialist services, and support resilience across diverse family and community systems [49, 53]. Peer-led or manualised interventions offer one route to scaling support for cascade testing and communication, particularly in resource-limited settings [62].

Cultural competence is central to inclusive family-oriented care. Black families and other historically excluded groups are under-represented in both clinical research and intervention studies [58], and models developed in Western, individualist contexts may sit uneasily alongside collectivist or relational decision-making traditions. Future models will need to integrate culturally grounded, multi-level strategies—community partnerships, peer-led initiatives, and generational health projects—that extend relational care beyond the consultation and foster trust in genomics across diverse communities.

#### Measuring family-oriented care

Building on Barbosa et al. [59] and Zhao et al. [18] findings, we believe research should prioritise outcomes that capture the complexity of family experiences. Frameworks such as FOCUS-GC [63] link counselling strategies to a broad range of outcomes, yet most existing measures remain focused on individual behaviours or short-term distress. Wade [64] similarly noted that current psychosocial metrics often fail to reflect the complex, long-term consequences of genetic information, especially in rare diseases, underscoring the need for theory-informed, systemic evaluation.

**Future priorities include**:

- Analysing clinical practice through process research—using observational and interactional studies to examine how family-oriented support is delivered in consultations and which counselling strategies underpin it.
- Broadening inclusion—capturing outcomes for the whole family system, including at-risk and not-at-risk relatives, children, men, partners, and step- or chosen family members.
- Embedding cultural competence—co-designing interventions and measures within diverse cultural and community groups to ensure relational congruence and responsiveness to different family structures and values.
- Strengthening ethical guidance for systemic practice—developing clear professional standards to navigate confidentiality, autonomy and family-level benefit when working systemically.
- Expanding condition scope—developing and testing interventions across a wider range of IGCs with varied trajectories, not only hereditary cancer syndromes.
- Strengthening evaluation—using longitudinal designs and family-level outcome measures aligned with FSI/FSGI phases to assess sustained adaptation over time.
- Innovating delivery—trialling community-based, retreat and virtual formats, and evaluating interdisciplinary and peer-supported models for feasibility and sustainability.

Collectively, these strategies signal a necessary shift toward relational, inclusive and context-sensitive models of care—ones that recognise families not only as recipients or transmitters of genetic information, but as dynamic systems requiring sustained, culturally attuned support across clinical and community settings.

#### Strengths and limitations of this review

This review systematically applied PRISMA-ScR and JBI guidance and, to our knowledge, is the first to synthesise this literature through a family systems lens [21]. A further strength is that the analysis was theory-informed: although, consistent with scoping review methodology, we did not undertake formal critical appraisal using a quality assessment tool, we did apply critical interpretive analysis, drawing on the FSI/FSGI frameworks to examine how family-oriented support was conceptualised and operationalised across the literature and to inform the heuristic continuum presented in Fig. 3. At the same time, the review has limitations. Evidence on clinical practice was derived largely from clinician self-report and was only minimally supplemented by observational or ethnographic work [23, 30], so the more nuanced counselling skills used by GHCPs may not be fully reflected in the published literature. However, our interpretation is supported by convergence with Barbosa et al.’s recent scoping review of process studies on family communication in genetic counselling [59], which approached the topic from a different analytical angle yet identified closely similar patterns: GHCPs consistently addressed family communication and provided informational resources, while psychosocial assessment, tailoring to family dynamics and sustained support remained less prominent and variable across contexts. This convergence across reviews increases confidence that these patterns reflect broader features of the literature rather than artefacts of our analytic approach alone. More broadly, the available literature was drawn predominantly from Western settings, which may limit the transferability of findings to other cultural and healthcare contexts where family roles, disclosure practices and decision-making norms differ. Unpublished community-based interventions may also have been missed, meaning that culturally grounded, locally developed or non-specialist models of family-oriented support are likely to be underrepresented.

## Conclusion

Family issues are increasingly recognised in genetic counselling, yet structured systemic approaches remain underdeveloped. Practice largely focuses on risk transmission, with limited psychosocial integration. Psychotherapeutic models show promise but limited scalability. Embedding FSI/FSGI principles into training, service design, and evaluation—centred on family-level adaptation—is essential. As genomic medicine becomes increasingly mainstream, more people with inherited genetic conditions or susceptibilities are likely to be identified outside specialist genetics services, often in settings where discussion of familial implications and relational support is more limited. Integrating family systems–informed approaches into routine care is therefore essential both to address the inherently shared nature of genetic information and to support psychosocial adaptation for families living with inherited conditions.

## Supplementary information

SUPPLEMENTARY MATERIAL 1

SUPPLEMENTARY MATERIAL 2

SUPPLEMENTARY MATERIAL 3

Video Presentation
