# Supplementary material for: Family-oriented support in genetic counselling: a scoping review of clinical practice and psychotherapeutic interventions
Source: Eur J Hum Genet. 2026 Jul 13;34(11):1622–43. doi: 10.1038/s41431-026-02165-z (PMC13743740; doi:10.1038/s41431-026-02165-z)
Supplement: Supplementary file 1 [file 41431_2026_2165_MOESM1_ESM.docx]

# Supplementary Material 1: Search Strategy and Analytic Framework

## 1. Search strategy

Comprehensive literature searches of electronic bibliographic databases were conducted by PhD researcher, Claire Green with support from experienced librarians and following McGowan et al. (2016) guideline statement. Prior to trial searches in Google scholar, collaborators (Alison Metcalfe, Marion McAllister and Mariangels Ferrer) were contacted for their subject knowledge, and signposted the researcher to key texts which informed initial search terms (Gaff and Bylund, 2010, Mcallister et al., 2020, MacLeod et al., 2021, Mendes et al., 2018).

The review acknowledges that prevailing models of genetic counselling are primarily derived from Western individualist contexts, and that the role of family may be more central in many cultural settings. Inclusion of studies across diverse cultural contexts was therefore encouraged, recognising the importance of culturally competent, family-oriented practice. Collaborators were contacted to access further grey literature. All forms of literature were included for screening including conference abstracts, commentaries, and opinion pieces.

A preliminary search was performed using Google Scholar and search terms were adjusted based on this search. The search strategy was peer reviewed by academic supervisor (Hilary Piercy) and librarians.

## Databases Searched

We systematically searched the following electronic databases:

1. PubMed/MEDLINE
2. PsycINFO
3. Embase
4. CINAHL
5. Web of Science
6. Scopus
7. Google Scholar (limited keyword search and forward citation chaining)

Search dates (from and to) 16^th^ March – 31^st^ March 2022. Searches were re-run, prior to the final analysis on 20^th^ August 2025.

Searches were restricted to English language and human studies.

Forward citation searches (screening articles that have cited any of the included articles) and backward citation searches (screening the reference list of included papers) were conducted on all articles that were included after full- text screening.

Six review articles exploring interventions in the context of genetic conditions were identified, three focused on family communication and uptake of testing in at-risk populations (Baroutsou et al., 2021, Zhao et al., 2022, Li et al., 2021) and two focused on psychosocial interventions (Barnett et al., 2022, Warner et al., 2021), with one focusing on support groups (Bertonazzi et al., 2022). Five were limited to hereditary cancer syndromes (Warner et al., 2021, Barnett et al., 2022, Baroutsou et al., 2021, Li et al., 2021, Bertonazzi et al., 2022). Although these papers were excluded from analysis, they were searched for family-oriented interventions.

## Search Rationale

The search strategy was developed using the Population–Concept–Context (PCC) framework, consistent with JBI guidance for scoping reviews. This framework provided a structured approach to defining the scope and focus of the review:

• Population: Families, probands, and relatives—explicitly or implicitly captured through family-related terms such as parent, child, partner, relative, and family communication. Health care professionals, including genetic counsellors and other genetic health care providers, were also included where studies explored their roles in facilitating family-oriented care.

• Concept: Approaches and practices incorporating a family component, including both clinical strategies and discrete psychotherapeutic or psychosocial interventions. Examples include narrative therapy, multifamily discussion groups, genogram and eco-map use, the Tree of Life approach, and psychoeducational or support-based models. These interventions were included where they aimed to enhance communication, adaptation, or coping within families affected by inherited genetic conditions.

• Context: Genetic and genomic counselling and clinical genetics services, encompassing both specialist and integrated models of care across hospital, community, and research settings.

We intentionally retained a genetics anchor to exclude unrelated family therapy literature. Searches applied no date restrictions, with limits to English language and human studies. Terms reflect both UK and US spelling variants (e.g., counselling/counseling; centred/centered). Both named systemic methods and broader psychosocial constructs (e.g., psychoeducation, family communication, family support) were included to capture evidence spanning standard clinical practice and discrete psychotherapeutic interventions.

## PubMed (MEDLINE) Search Strategy

The following search strategy was applied in PubMed and adapted for use in other databases:

Search: **("Genetic Counseling"[Mesh] OR "Genetic Services"[Mesh] OR "genetic counsel*"[tiab] OR "genomic counsel*"[tiab] OR "genetic counsell*"[tiab] OR "genomic counsell*"[tiab] OR "genetic clinic*"[tiab] OR "clinical genetic*"[tiab] OR "genomic medic*"[tiab] OR ("genetic*"[tiab] AND (risk[tiab] OR condition*[tiab] OR disease*[tiab])) ) AND ( "Family Therapy"[Mesh] OR "Family Relations"[Mesh] OR "narrative therap*"[tiab] OR "systemic therap*"[tiab] OR "family therap*"[tiab] OR "couple therap*"[tiab] OR "multifamily"[tiab] OR "tree of life"[tiab] OR "genogram*"[tiab] OR "family orient*"[tiab] OR "family focus*"[tiab] OR "family-centred"[tiab] OR "family-centered"[tiab] OR "family communicat*"[tiab] OR "family support"[tiab] OR "supporting famil*"[tiab] ) AND (english[lang]) NOT (animals[mh] NOT humans[mh])**

## Adaptations for Other Databases

PsycINFO: Subject headings mapped to equivalent APA Thesaurus terms (e.g., “Genetic Counseling”, “Family Therapy”).

Embase: Emtree terms applied alongside free-text (e.g., 'genetic counseling'/exp, 'family therapy'/exp).

CINAHL: CINAHL Subject Headings substituted where available.

Web of Science/Scopus: Keyword-based searches only, using TITLE-ABS-KEY fields.

Google Scholar: Key phrases searched (e.g., “genetic counseling family therapy”, “genetic counselling narrative therapy”), first 200 results screened, with forward citation chasing of key included papers.

2. **Application of the FSI/FSGI‑informed analytic framework**

To assess how publications engaged with family‑oriented care, we applied four FSI/FSGI‑informed analytic dimensions: illness/genetic‑condition characteristics, family‑system processes, psychosocial context, and care structure and delivery. These dimensions were used interpretively to examine how far each publication moved beyond proband‑centred risk communication toward systemic family‑oriented care. This was not a formal scoring exercise; rather, the framework was used to distinguish between studies that merely recognised the familial implications of genetic risk and those that actively engaged with family relationships, psychosocial adaptation, and evolving needs over time.

The fourth dimension, care structure and delivery, was particularly important because it captured whether FSI/FSGI principles were operationalised in practice. We considered who was involved in care, when support occurred, how intensive or structured the support was, whether relatives were directly included, and whether support was reactive, optional, patient‑initiated, or embedded within care pathways.

**Stage 1 example: Forrest et al. [24]**

This study illustrates predominant Stage 1 practice. Genetic risk is framed primarily around identifying at‑risk relatives and encouraging the communication of risk information. Family‑system processes are acknowledged, but largely through the proband’s responsibility to disclose information. Relatives are generally positioned as recipients of risk information rather than as active participants in care. Psychosocial context is present but indirect: emotional readiness, family barriers, and practical difficulties may be recognised, but support typically depends on patients or relatives proactively seeking help. Care remains proband‑mediated, relying on consultations, contact details, summary letters, family letters, and patient‑initiated follow‑up. This reflects Stage 1 engagement, in that the family is genetically relevant but not systematically engaged as the unit of care.

**Stage 2 example: Peters et al. [42,43]**

These studies illustrate a Stage 2–3 bridge. Genetic risk is situated within the individual’s family and social context through CEGRM/pedigree‑based assessment. Unlike standard pedigree completion, this approach uses the pedigree as a relational and psychosocial tool, mapping information exchange, emotional support, practical support, and social relationships. Psychosocial context is actively elicited, including support networks, emotional exchanges, and the wider social milieu. However, care structure remains primarily consultation‑based and adjunctive. The tool deepens assessment and may itself have therapeutic value, but wider family members are not routinely engaged as participants within an ongoing systemic care pathway. This positioning fits a Stage 2–3 bridge, as it moves beyond biomedical risk documentation without fully constituting embedded systemic family care.

**Stage 3 example: SPRinG / Eisler et al. [44]**

This study illustrates Stage 3 systemic family‑oriented care in both orientation and structure, while remaining a feasibility intervention rather than routine longitudinal care. Genetic risk is conceptualised as an ongoing family experience rather than a discrete testing event. The multifamily group format makes visible that different relatives may be at different stages of understanding, emotional adjustment, and life‑stage need. Family‑system processes are central: parents, children, and young people are supported to discuss communication, coping, and relational challenges together. Psychosocial context is explicitly integrated through attention to family strengths, isolation, emotional adjustment, shared learning, and adaptation. Care is structured as a facilitated multifamily intervention delivered by trained professionals, directly engaging families as the focus of support. It therefore approximates Stage 3, as it treats the family system as the target of care and employs systemic methods, while also highlighting an implementation gap: sustained longitudinal delivery is not yet embedded within routine care pathways.
